# Impact of Co-Supplementation of Gallic Acid With Vitamin C on Oxidant Stress, Inflammation, Hepatic and Renal Histology of Type 2 Diabetic Rats Induced With Fructose and Streptozotocin

**DOI:** 10.33549/physiolres.935785

**Published:** 2026-06-01

**Authors:** Shufang YAN, Xu XIANG, Wei HUA

**Affiliations:** 1 Department of Endocrinology, Huaihe Hospital of Henan University, Kaifeng, China; 2 Department of Nephrology, The First Affiliated Hospital of Chongqing Medical and Pharmaceutical College, Chongqing, China

**Keywords:** Antioxidants, Type 2 diabetes mellitus, Diabetic liver disease, Diabetic kidney complication, Natural products

## Abstract

This study assessed the impact of gallic acid plus vitamin C on systemic oxidant stress, inflammation, hepatic and renal histology of rats with type 2 diabetes mellitus. Twenty five albino male rats were allotted into five categories of five rats each and treated hereafter as: control and diabetic group (fed rat diets and water), vitamin C (diabetic treated rats administered vitamin C, 50 mg/kg), gallic acid (diabetic treated rats administered gallic acid at 20 mg/kg), gallic acid plus vitamin C (diabetic treated rats administered 20 mg/kg gallic acid and 50 mg/kg vitamin C). The study duration was forty two days. The diabetic group had significant raise (P<0.05) of fasting glucose, glycated hemoglobin, insulin resistance scores, oxidative stress and inflammatory markers (in the liver, kidney and serum), alteration of relative liver and kidney weights, liver and kidney function indices, significant decrease (P<0.05) in total hemoglobin, serum insulin, insulin sensitivity and pancreatic beta cell function scores, body weights and pathological changes in their liver and kidney histology. These changes were ameliorated following supplementation of the diabetic group with gallic acid, vitamin C and gallic acid plus vitamin C combined treatment. Intervention with vitamin C was more efficacious than gallic acid. However, combined treatment of gallic acid plus vitamin C was more efficacious than the single treatments in modulating systemic and local oxidative stress, inflammation and in restoring the altered liver and kidney histology of the diabetic rats.

## Introduction

Diabetic nephropathy (DN) is amongst the complexities of type 2 diabetes mellitus (T2DM) and it can cause vascular diseases in diabetic patients [1]. Current statistics revealed that nearly 30 to 40 % of T2DM patients globally develop DN and DN contributes to the mortality of these patients. In addition to DN, diabetic patients suffer from several liver disorders such as: metabolic dysfunction-associated steatotic liver disease (formerly known as nonalcoholic fatty liver disease), cirrhosis and others that contribute to the mortality of diabetic patients [2].

Oxidative stress (local and systemic) has been considered as a contributory factor to the emergence of diabetes and its complications [3]. Enhanced generation of reactive oxygen species during oxidative stress activates pro-inflammatory pathways through nuclear factor kappa B (NF-κB) activation, leading to chronic inflammation and diabetic complications [4].

Owing to the involvement of oxidative stress in the emergence of diabetes and its complications, the side effects of the currently recommended drugs for T2DM management together with the lesser complexities of natural antioxidants (compared with synthetic anti-diabetic drugs), there has been a paradigm shift of attention towards the use of natural antioxidants to treat T2DM and prevent its progression to complications [3].

Gallic acid is one of such natural antioxidants with proven usefulness in attenuating oxidative stress linked diseases. GA is found in many dietary substances, such as vegetables, fruits, etc. Some of the biological properties of gallic acid are: antioxidant, anti-inflammatory, anti-obesity and anti-tumor properties [5].

Vitamin C is a water-soluble antioxidant vitamin that neutralizes free radicals by acting as a reducing agent in hydroxylation reactions [6]. Vitamin C is found naturally in several fruits and vegetables [7]. Some of the pharmacological benefits of vitamin C include: antioxidant [7], anti-inflammatory properties [8] and others.

Although earlier investigators [5,8] documented the anti-hyperglycemic effects of gallic acid and vitamin C, the possibility of combining them to mitigate diabetic liver and kidney complications has not been reported in literature. In light of the increasing global prevalence of diabetic liver and kidney complications despite current therapies for diabetes management, it becomes necessary to conduct more researches into alternative or complementary therapies as recommended by the American Diabetic Association [9] in order to avert the emergence of complications of DM. This study thus reported the consequence of combining gallic acid with vitamin C on systemic oxidant stress, inflammation, hepatic and renal histology of fructose/streptozotocin (STZ) instigated type 2 diabetic rats.

## Materials and Methods

### Chemicals and kits

Aspartate aminotransferase (AST), alanine aminotransferase (ALT) and alkaline phosphatase (ALP) kits were purchased from Randox Laboratory (United Kingdom.). Gallic acid, as well as fructose, STZ, trisodium citrate dehydrate, in addition to citric acid were purchased from Sigma-Aldrich (U.S.A). Assay kits (ELISA) for NF-κB and tumor necrosis factor (TNF) α were purchased from Elabscience (U.S.A). Additional chemicals and reagents that we used were of high grade and they were purchased from well-known and reliable suppliers.

### Experimental animals

Forty five male albino rats (Wistar strain, six weeks old) were used. Upon arrival, the rats were acclimated for seven days under standard laboratory condition (12-hour light/dark cycle and temperature of 22–25 °C). They were housed in clean, stainless cages and fed growers mash and tap water ad libitum.

The animal procedure complied with the guideline by the National Institute of Health [9] for care and usage of laboratory animals.

### Induction of T2DM

T2DM was induced in the rats using a dual model (fructose administration and STZ injection) that produces insulin resistance (IR) and β-cell dysfunction similar to human T2DM [10].

All groups except the control, received 10 % fructose solution as their drinking water for 14 days to induce IR. The solution was freshly prepared daily before administration to the rats. On the evening of day 14, the rats were fasted (14 h) and the succeeding day, their threshold blood glucose determinations were carried out. Next, the rats (except the control) received an intraperitoneal injection of STZ (40 mg/kg body weight) as a single dose, dissolved in fresh sodium citrate buffer (0.1 M, pH 4.5). After 72 h, fasting blood glucose was measured from their tail vein using a glucometer. Rats that showed fasting blood glucose levels ≥200 mg/dl were classified as having T2DM [10].

### Experimental design

After confirming T2DM, the control and diabetic animals were randomly allocated into five groups (n=5 for each group) and tended thus:

Group 1 (control) was administered commercial rat diets and tap water daily
Group 2 (diabetic untreated) was administered commercial rat diets (finisher feeds in pellets) and tap water daily
Group 3 (diabetic treated 1) was administered rat diets and vitamin C at 50 mg/kg bwt
Group 4 (diabetic treated 2) was administered rat diets and gallic acid at 20 mg/kg bwt
Group 5 (diabetic treated 3) was administered rat diets, gallic acid at 20 mg/kg bwt as well as vitamin C at 50 mg/kg.

The interventions were given orally and they were dissolved in tap water before intake by the rats. The dose which we utilized for gallic acid and vitamin C in this research was derived from earlier investigators as their therapeutic doses [11–12].

The rats in the above groups received their diets and water *ad libitum* and their weights were taken throughout the treatment period. Upon completion of six weeks of treatments, each rat was fasted and the day after, blood were obtained from their tail prick and glucose levels in their blood were evaluated with a glucose meter. Thereafter, rats were tranquilized with 90 mg/kg of ketamine and blood were gotten from their retro orbital veins into heparin tubes for total hemoglobin (Hb) and glycated hemoglobin (HbA1c) assays. Serum was collected in plain tubes for assay of insulin, AST, ALT, ALP, urea, creatinine, uric acid, serum total proteins, albumin, superoxide dismutase (SOD), glutathione peroxidase (GPx), glutathione-S-transferase (GST), reduced glutathione (GSH), malondialdehyde (MDA), NF-κB, TNF-α and C-reactive protein (CRP) concentrations.

### Tissue analysis

The liver and kidneys of the rats were removed, blotted and measured for calculation of their relative weights. Thereafter, the tissues were halved with a part kept inside 10 % neutral-buffered formaldehyde for histological analysis while the remaining part was analyzed for oxidative stress and inflammatory markers.

Serum insulin, homeostatic model assessment of insulin resistance (HOMA-IR), homeostatic model assessment of pancreatic beta cell function (HOMA-β) and quantitative insulin sensitivity check index (QUICKI) determinations.

Serum insulin was estimated using commercial assay kits (Monobind Inc.) using ELISA kits [13]. Results were stated as mU/ml. HOMA-IR calculation was done as: HOMA-IR = [Serum insulin (μU/ml) multiplied by fasting blood glucose (mmol/l)/22.5] [14–15]. HOMA-β calculation was done as: HOMA-β = {20 × Insulin (μIU/l)/glucose (mmol/l)} − 3.5 while QUICKI was calculated as: QUICKI = 1/[log fasting glucose (mmol/l) + log fasting serum insulin (μU/ml)] [15].

### Assessment of liver function indices in the serum

Activities of AST, ALT and ALP were evaluated with assay kits (Agappe) while results were written as units/l.

### Assay of renal function markers

Serum total proteins as well as albumin, urea, creatinine and uric acid were evaluated with Agappe assay kits. Results for total proteins and albumin were written as g/dl while results for urea, creatinine and uric acid were written as mg/dl.

### Measurement of lipid peroxidation marker as well as antioxidant indices in the serum and tissues

The marker of peroxidation of lipids (MDA) was quantified using the methodology of Buege and Aust [16]. SOD activity was evaluated with the methodology of Sun and Zigma [17]. GPx activity was quantified following the protocol of Rotruckjt *et al*. [18]. GST activity was evaluated employing the methodology of Habig *et al*. [19]. GSH concentration was assessed according to the protocol of Sedlak and Lindsay [20]. Results were stated as units/ml for SOD, GPx and GST, μmol/ml for GSH and MDA in the serum; units/mg protein for SOD, GPx and GST, μmol/mg protein for GSH and MDA in the tissues.

### Measurement of inflammatory markers’ levels in the serum and tissues

NF-κB and TNF-α concentrations were assessed using their assay kits following the instructions of their manufacturers. CRP was evaluated using its assay kit (Monobind Inc). Results were stated as ng/ml for NF-κB and CRP and pg/ml for TNF-α in the serum; pg/mg protein for NF-κB and pg/g protein for TNF-α in the tissues. All biochemical assays were carried out using Enzyme Linked Immunosorbent Assay (ELISA) microplate reader.

### Statistical analysis

Statistical analysis was performed using a statistical software (SPSS, version 17.0). We presented our data as means plus standard deviations. Analysis of variance (one way) and Duncan multiple range tests were utilized for comparison of mean values and *post hoc* tests. We plotted our graphs using Graph Pad Prism (Version 6.0). Data were considered to be significant at P<0.05.

## Results

### Gallic acid plus vitamin C combined treatments’ effect on glucose, insulin values, HOMA-IR, HOMAβ and QUIKCI scores of rats

**Fig. 1:**
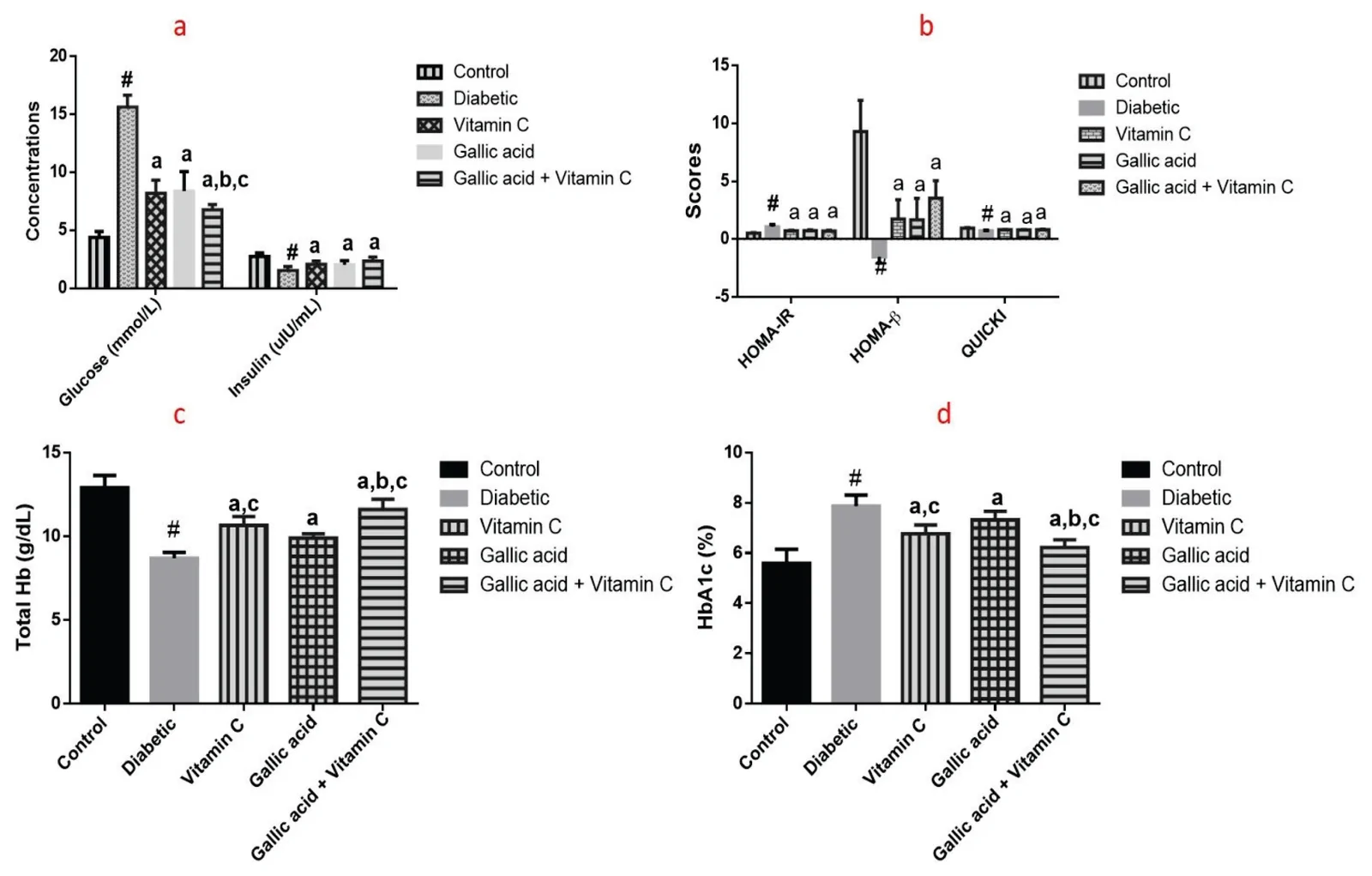
(**a–d**). Impact of gallic acid and vitamin C on blood glucose, serum insulin, homeostatic model assessment of insulin resistance, pancreatic β-cell function, quantitative insulin sensitivity check index, total and glycated hemoglobin concentrations of rats with T2DM. Results were reported as means ± standard deviations. ^#^ statistically significant (P<0.05) compared with the control. ^a^ statistically significant (P<0.05) compared to the diabetic; ^b^ statistically significant (P<0.05) compared to vitamin C; ^c^ statistically significant (P<0.05) compared to gallic acid; HOMA-IR – homeostatic model assessment of insulin resistance; HOMA-β – homeostatic model assessment of pancreatic beta cell function; QUICKI – quantitative insulin sensitivity check index. Total Hb – Total hemoglobin; HbA1c – Glycated hemoglobin.

As displayed in Figure 1a and b, remarkable increases (P<0.05) in glucose, and HOMA-IR were obtained for the diabetic rats unlike the control group. Additionally, the diabetic group presented distinctly lower (P<0.05) insulin levels, HOMA-β and QUICKI scores unlike the control. These changes were reversed with notable reduction (P<0.05) of glucose levels as well as HOMA-IR scores but increased (P<0.05) insulin, HOMA-β and QUICKI scores of the diabetic rats intervened with gallic acid and vitamin C as single and combined therapies. Moreover, intervention using gallic acid plus vitamin C produced distinctly lower (P<0.05) glucose levels and significantly higher (P<0.05) insulin levels contrasted with gallic acid or vitamin C interventions.

### Effect of gallic acid and vitamin C combined treatment on total Hb and HbA1c concentrations of rats

As displayed in Figure 1c and d, notable attenuation (P<0.05) of total Hb concentrations but elevations (P<0.05) in HbA1c concentrations were seen in the diabetic rats contrasted with the control group. Upon intervention with gallic acid, vitamin C or their combined treatment, the diabetic treated animals achieved remarkable increases (P<0.05) in total Hb concentrations with reductions (P<0.05) in HbA1c concentrations contrasted with the diabetic group. Moreover, vitamin C supplementation produced significantly higher (P<0.05) total Hb concentrations and significantly lower (P<0.05) HbA1c concentrations compared to gallic acid supplementation whilst the combinatorial therapy produced significantly higher (P<0.05) total Hb concentrations and significantly lower (P<0.05) HbA1c concentrations contrasted to intervention with gallic acid as well as vitamin C.

### Body weights and relative organ weights of rats

**Fig. 2:**
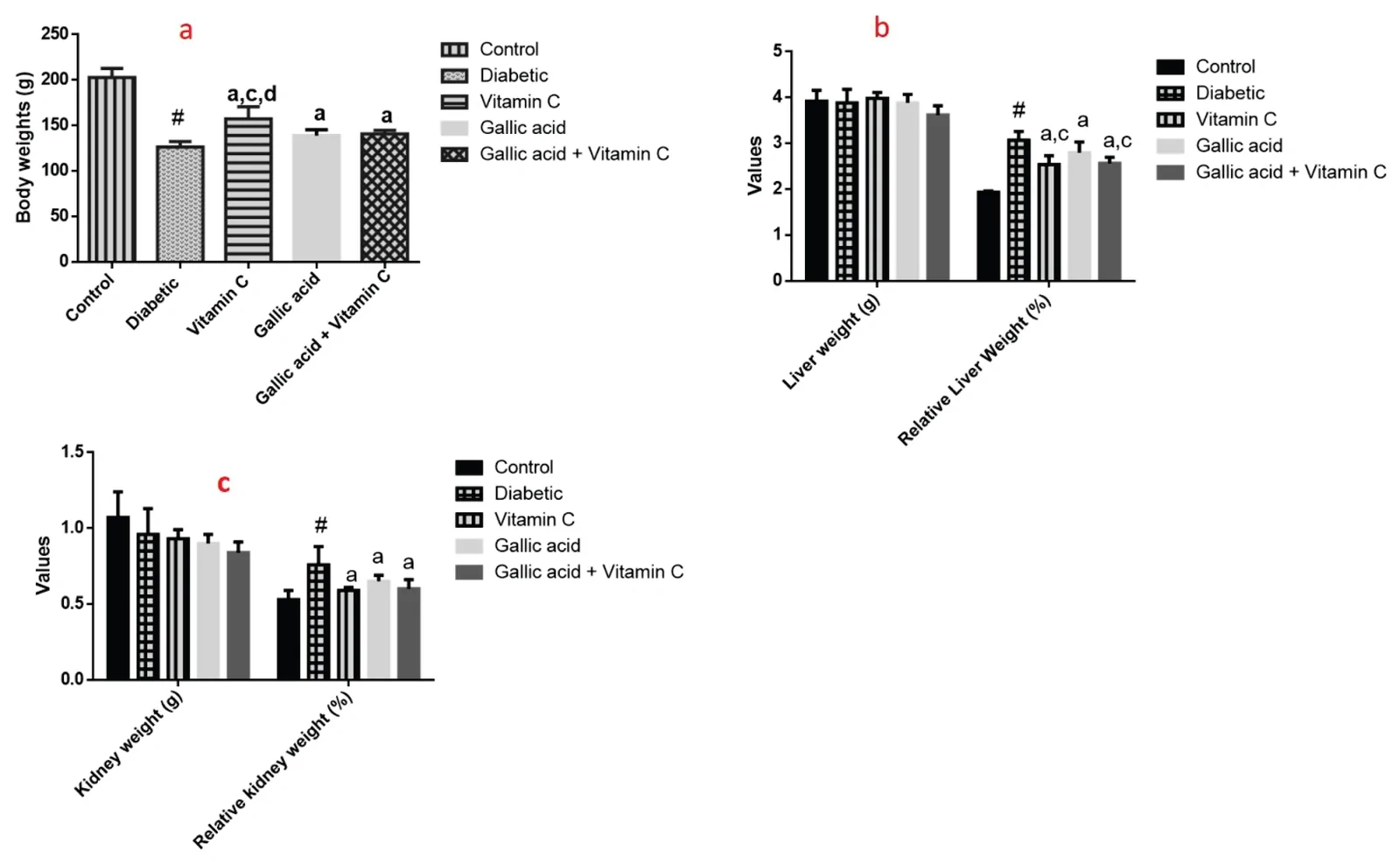
(**a–c**). Impact of gallic acid and vitamin C on body weights, liver and kidney weights and relative liver and kidney weights of rats with T2DM. Results were reported as means ± standard deviations. ^#^ statistically significant (P<0.05) compared to the control. ^a^ statistically significant (P<0.05) compared to the diabetic; ^c^ statistically significant (P<0.05) compared to gallic acid; ^d^ statistically significant (P<0.05) compared to gallic acid + Vitamin C.

As manifest in Figure 2a, the weight of the diabetic untreated rats appeared profoundly diminished (P<0.05) contrasted with the control. Upon intervention with gallic acid or vitamin C and the combined treatment, there were remarkable weight gains (P<0.05) in the diabetic treated animals contrasted with the diabetic group. In addition, combined treatment of gallic acid and vitamin C produced distinctly lower (P<0.05) glucose levels and significantly higher (P<0.05) insulin levels contrasted to intervention with gallic acid or vitamin C. Further, vitamin C achieved better weight gain (P<0.05) in the diabetic rats contrasted to intervention with gallic acid or the combined treatment.

As revealed in Figure 2b and Figure 2c, the relative liver and kidney weights of the diabetic rats were markedly elevated (P<0.05) in contrast to the diabetic group. These changes were remarkably attenuated (P<0.05) upon supplementation of the diabetic animals with gallic acid, vitamin C or their combined treatment. Further, vitamin C or the combined treatment achieved better reduction of the elevated relative liver weights of the diabetic rats contrasted to gallic acid treatment.

### Effect of the combined treatment on antioxidant indices and lipid peroxidation marker in rats

**Fig. 3:**
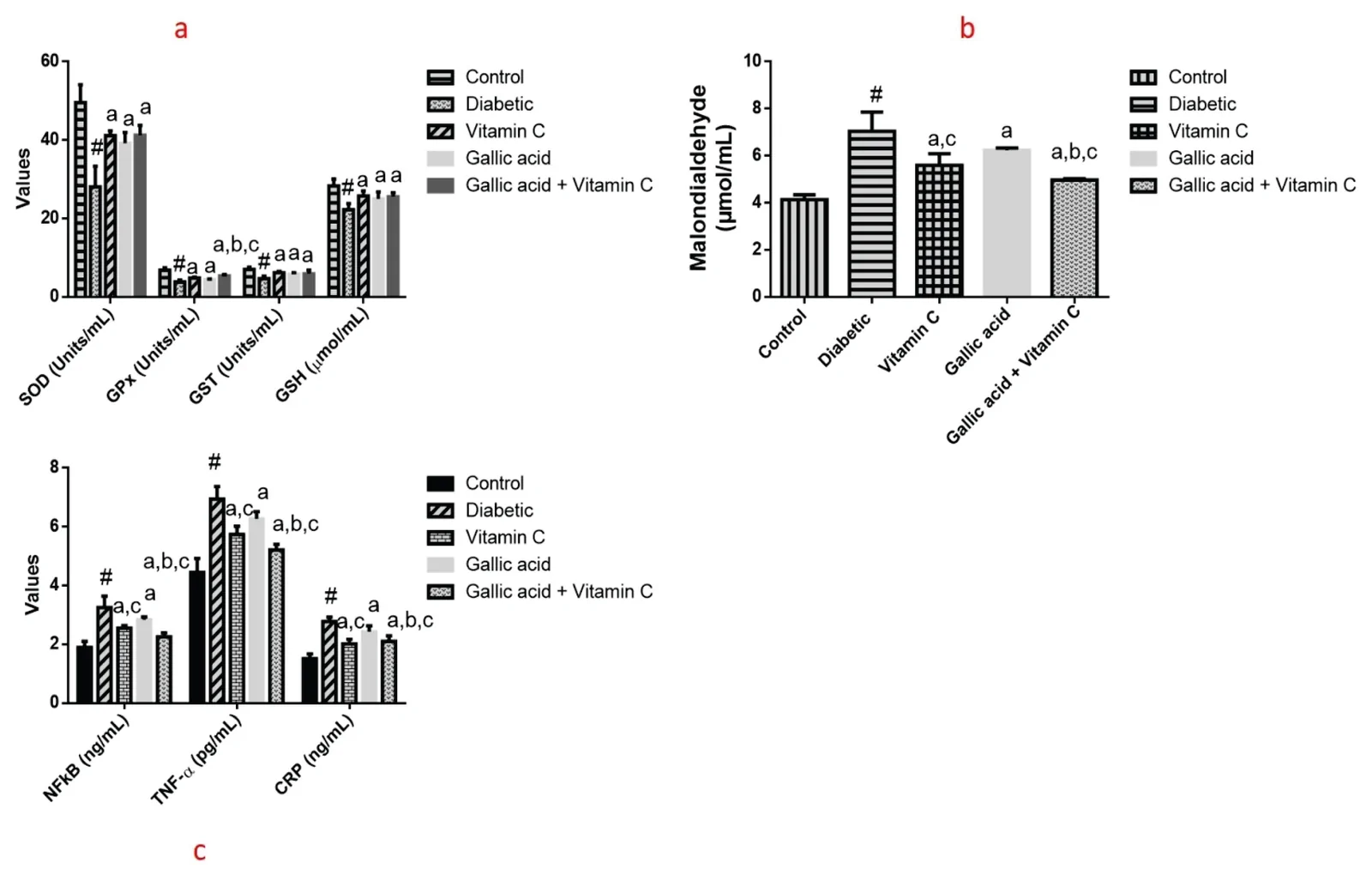
(**a–c**). Effect of gallic acid and vitamin C on antioxidants, oxidative stress and inflammatory markers in the sera of rats with T2DM. Results were reported as means ± standard deviation. ^#^ statistically significant (P<0.05) compared to the control. ^a^ statistically significant (P<0.05) compared to the diabetic; ^b^ statistically significant (P<0.05) compared to vitamin C; ^c^ statistically significant (P<0.05) compared to gallic acid. SOD – Superoxide dismutase; GPx – Glutathione peroxidase; GST – Glutathione-S-transferase; GSH – reduced glutathione; MDA – Malondialdehyde; NF-κB – Nuclear factor kappa B; TNF-α – Tumor necrosis factor alpha; CRP – C-reactive protein.

Table 1 and Figure 3a-b reveal the impact of the combined treatment on the tissue and serum levels of antioxidant indices and lipid peroxidation marker in rats with T2DM. Significant decline (P<0.05) in GPx, GST (in the serum), SOD activities and GSH concentration with remarkable elevation (P<0.05) of MDA level was obtained in the liver, kidney and sera of the diabetic rats compared to the control. Following supplementation of the diabetic rats with the interventions as single or combined treatments, there were notable reduction (P<0.05) of MDA levels with notable raises (P<0.05) in GPx, GST (in the serum) and SOD activities (except in the liver of gallic acid group) and GSH concentration of the diabetic animals compared to the diabetic group. Moreover, vitamin C supplementation achieved better reduction (P<0.05) of the MDA concentrations in the sera and kidney of the diabetic animals contrasted to gallic acid supplementation whilst the combined treatment showed a more efficient reduction (P<0.05) of MDA level (in the serum liver and kidneys of the rats) and better elevation (P<0.05) of GPx activity than either gallic acid or vitamin C in the serum and liver of the rats.

### Effect of the combined treatment on serum levels of inflammatory indicators of rats

Table 2 and Figure 3c reveals the impact of gallic acid and vitamin C interventions on the tissue and serum levels of inflammatory indicators in rats with T2DM. Remarkable elevations (P<0.05) of NF-κB and TNF-α (in the liver, kidney and tissues) and CRP levels were found in the diabetic animals contrasted with the diabetic group. Upon administration of the diabetic animals with the interventions as single or combined treatments, there were remarkable decline (P<0.05) in the NF-κB, TNF-α and CRP levels of the diabetic animals compared to the diabetic group. Also, vitamin C supplementation achieved better reduction (P<0.05) of NF-κB and TNF-α (in the liver, kidney and serum) as well as CRP concentrations of the diabetic rats compared to gallic acid supplementation but the combined treatment reduced (P<0.05) the NF-κB and TNF-α concentrations of the diabetic animals contrasted with gallic acid or vitamin C. Further, the combined treatment remarkably decreased (P<0.05) the CRP levels of the diabetic animals contrasted with gallic acid supplementation but not with vitamin C supplementation.

### Impact of the combined treatment on liver function indices of rats

**Fig. 4:**
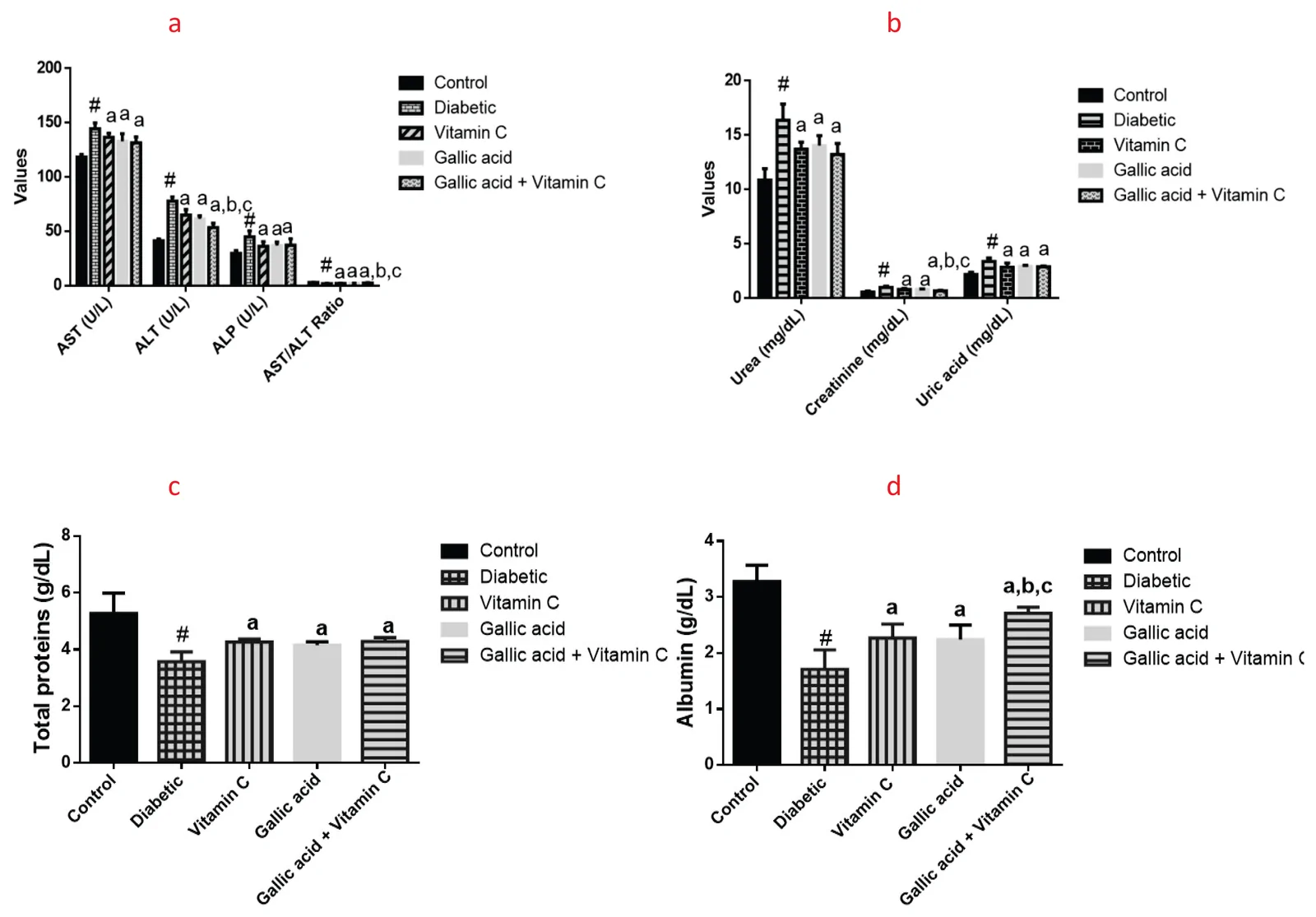
(**a–d**). Impact of gallic acid and vitamin C on serum liver and kidney function markers of rats with T2DM. Results were reported as means ± standard deviation. ^#^ statistically significant (P<0.05) compared to the control. ^a^ statistically significant (P<0.05) compared to the diabetic; ^b^ statistically significant (P<0.05) compared to vitamin C; ^c^ statistically significant (P<0.05) compared to gallic acid. AST – Aspartate amino transaminase; ALT – Alanine amino transaminase; ALP – Alkaline phosphatase.

Figure 4a presents the impact of gallic acid plus vitamin C combined treatment on the serum concentrations of liver function indices in rats with T2DM. Notable elevation (P<0.05) of AST, ALT and ALP activities and notable attenuation (P<0.05) of AST/ALT ratio were observed in diabetic rats in contrast to the control. Upon supplementation of the diabetic rats with gallic acid, vitamin C and their combined treatment, there were remarkable decline (P<0.05) in their serum AST, ALT and ALP activities with notable elevations (P<0.05) of their AST/ALT ratios contrasted to the diabetic group. Furthermore, the combined treatment distinctly decreased (P<0.05) the ALT activities of the diabetic rats while it remarkably raised (P<0.05) their AST/ALT ratios contrasted to gallic acid or vitamin C interventions.

### Effect of the combined treatment on kidney function indices of rats

Figure 4b to 4d show the impact of combined treatment of gallic acid and vitamin C on the kidney function indices in the sera of rats with T2DM. Significant elevation (P<0.05) of urea, creatinine and uric acid levels and significant decline (P<0.05) in total protein and albumin levels were obtained in the diabetic rats compared to the control. Upon supplementation of the diabetic rats with gallic acid, vitamin C and their combined treatment, there were remarkable decline (P<0.05) in their serum urea, creatinine and uric acid levels but significant elevation (P<0.05) of their total proteins and albumin levels compared to the diabetic group. In addition, the combined treatment remarkably decreased (P<0.05) the creatinine levels of the diabetic animals while it notably surged (P<0.05) their albumin levels in comparison to interventions with gallic acid or vitamin C.

### Histological assessment of the liver and kidneys of rats

**Fig. 5:**
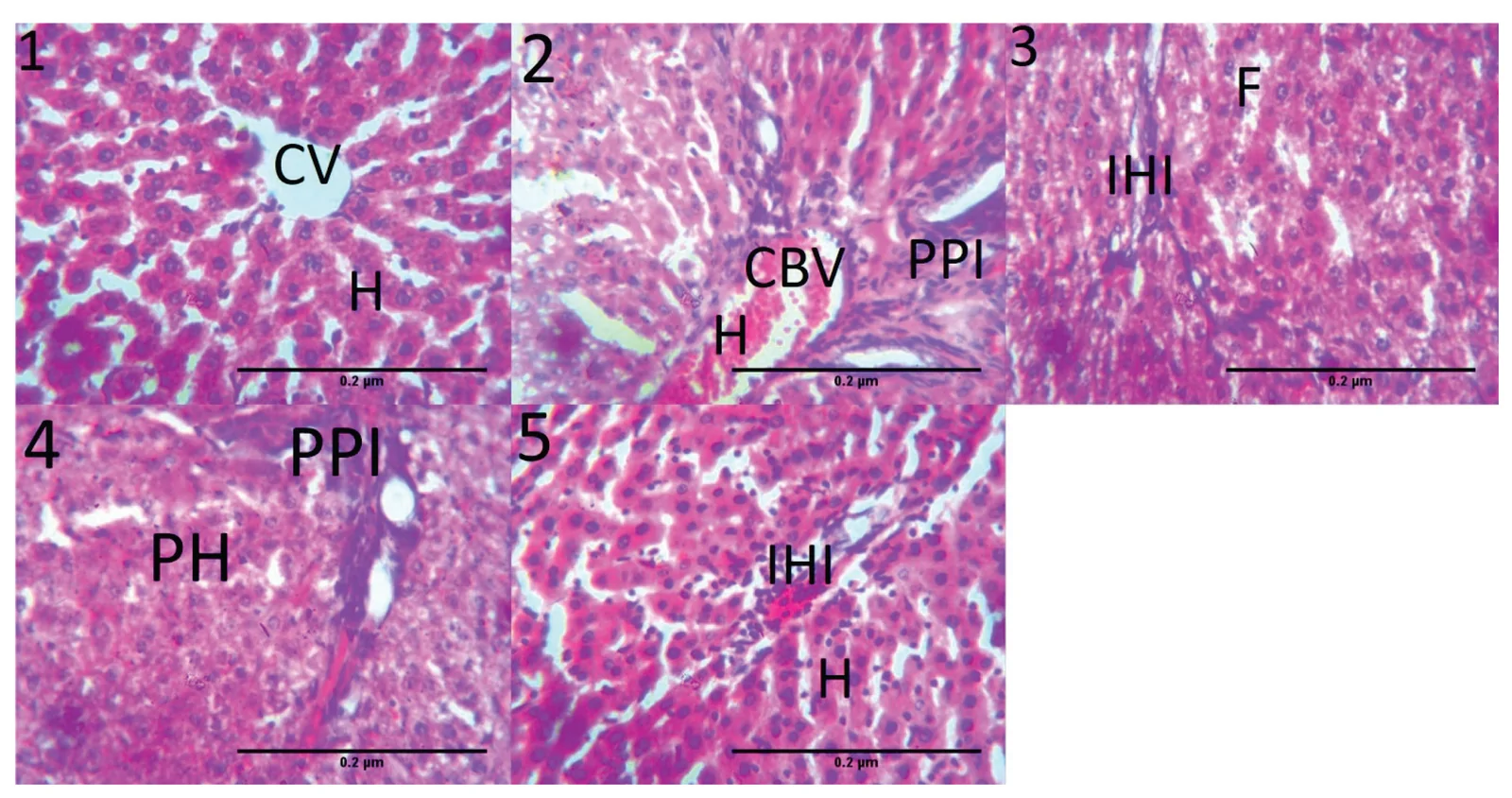
Impact of gallic acid and vitamin C on the histopathology of the liver of type 2 diabetic rats. Magnification: ×400 (H&E). 1 (Control); 2 (Diabetic); 3 (Vitamin C); 4 (Gallic acid); 5 (Gallic acid + Vitamin C).

Figure 5 reveals the impact of gallic acid and vitamin C on the hepatic histology of rats with fructose/STZ T2DM model. (1) Control group histology showed well perfused normal hepatic tissue with central vein and active hepatocytes. (2) The diabetic group histology presented severe alteration with severe aggregate of periportal inflammation, congestion of blood vessels and hemorrhage. (3) Vitamin C group histology presented mild aggregate of intra hepatic inflammation and mild fatty changes. (4) Gallic acid group histopathology presented severe aggregate of periportal inflammation with pyknotic hepatocyte. (5) Gallic acid/Vitamin C group histology revealed active hepatocytes with moderate aggregate of intra hepatic inflammation.

**Fig. 6:**
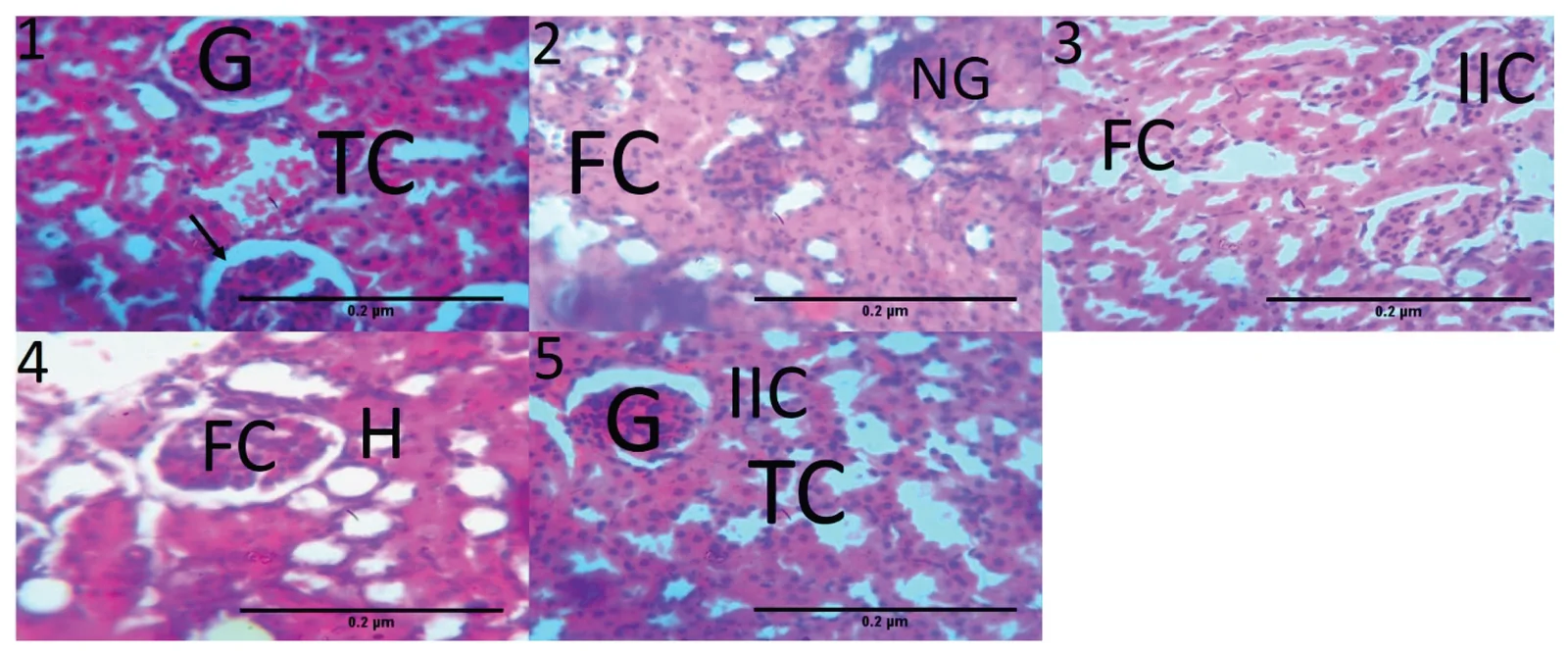
Impact of gallic acid and vitamin C on the histopathology of the kidney of type 2 diabetic rats. Magnification: ×400 (H&E). 1 (Control); 2 (Diabetic). 3 (Vitamin C); 4 (Gallic acid); 5 (Gallic acid + Vitamin C).

Figure 6 reveals the impact of gallic acid and vitamin C combined treatment on the renal histology of rats with fructose/STZ T2DM model. (1) Control histology revealed active renal tissue with glomeruli and active tubular cells. (2) Diabetic group histology revealed severe deterioration of their kidney with severe fatty changes with hemorrhagic and necrotic glomeruli. The overall features were indicative of chronic nephrititis. (3) Vitamin C group histology revealed renal tissue that had mild infiltration of inflammatory cells and mild fatty changes. (4) Gallic acid group histology revealed renal tissue with severe fatty changes and hemorrhage. (5) Gallic acid/Vitamin C group histology showed renal tissue with active tubular cell and mild inflammatory cells.

## Discussion

HOMA-IR, HOMA-β and QUICKI scores are widely utilized to measure the degree of glycemic control, insulin sensitivity, resistance, β-cell function and glucose-insulin balance [21–23]. The attenuated QUICKI and HOMA-β scores plus the elevated HOMA-IR index and glucose levels of the diabetic animals implies fructose/STZ administration triggered reduced insulin sensitivity, increased insulin resistance, altered functionality of pancreatic β-cells as well as hyperglycemia in them.

The increased QUICKI scores, reduced HOMA-IR indices, elevated HOMA-β scores and the diminished levels of glucose in the blood of the diabetic animals who received gallic acid, vitamin C, plus their combined treatment, suggests that the interventions attenuated hyperglycemia in the rats possibly through enhancement of insulin sensitivity and repair of altered pancreatic β-cells. Similar reports on the insulin sensitizing actions of gallic acid and vitamin C were given by Patel *et al*. [24] and Bunbupha *et al*. [25] respectively.

We demonstrated that the combined treatment controlled hyperglycemia better than the single treatments. This was seen from the attenuated levels of blood glucose along with the raised serum insulin concentrations of the diabetic rats who received the combined treatment contrasted with the diabetic rats who received the single treatments.

During chronic hyperglycemia, a number of proteins including hemoglobin undergo non-enzymatic glycation, triggering complications [11,23]. Protein glycation may explain the decreased total Hb but increased HbA1c concentrations of the diabetic rats. HbA1c analysis has proven to be relevant in monitoring the effectiveness of interventions for T2DM control [23]. This is because HbA1c blood level reflects glycemia over a longer period and its value is not affected by recent food intake. The increased HbA1c concentration of the diabetic animals in this study, implies their poor glycemic control. We also revealed that gallic acid, vitamin C and the combined treatment revamped the glycemic status of the diabetic animals. This became apparent from the attenuated HbA1c status of the diabetic rats which were given the single and combined treatment. However, vitamin C was superior to gallic acid in achieving glycemic control while the combined therapy demonstrated superiority to the single therapies in achieving glycemic control. This was evidenced from the attenuated HbA1c status of the diabetic animals which took vitamin C in comparison with the diabetic animals which were given gallic acid as well as the attenuated HbA1c status of these diabetic rats which took the combined treatment in comparison with the HbA1c status of these diabetic rats which took either gallic or vitamin C.

During chronic hyperglycemia, IR prevents the uptake of glucose into the peripheral tissues for conversion to energy [26]. As a result, the body burns stored fat and muscle proteins to generate energy, leading to weight loss which we saw in the diabetic rats of this study [26]. The weight gain that was obtained for those diabetic rats which took gallic acid, vitamin C and the combined treatment could be ascribed to attenuation of hyperglycemia by these interventions. The lower weight gain of those diabetic rats which took gallic acid and the combined treatment in comparison with the diabetic rats which took vitamin C, could be attributed to the weight reducing properties of polyphenols, one of which is gallic acid [27] and not because of poor control of glycemia because the combined treatment controlled glycemia better than vitamin C in this study.

Changes in organ weights are early indicators of pathological changes to organs [28]. Therefore, the elevated relative liver and kidney weights of the diabetic rats suggest pathological changes to these organs. However, the reduced relative weight of the liver as well as relative weight of the kidney of the diabetic rats which took gallic acid, vitamin C and the combined treatment, implies the ability of the interventions to abolish pathological changes to the liver and kidney brought on by fructose/STZ challenge in rats.

The decline of the relative weight of the liver of the diabetic rats which were intervened with vitamin C and the combined treatment (as against gallic acid group) implies that vitamin C plus its co-treatment with gallic acid, abolished remarkably, the alterations in the liver of the diabetic rats in a way that was better than gallic acid intervention.

The cytotoxic action of oxygen free radicals is exerted on membrane lipids, leading to their peroxidation and generation of MDA as one of the products [26]. SOD catalyzes the dismutation of superoxide radicals, with subsequent formation of hydrogen peroxide and molecular oxygen [26,29]. The hydrogen peroxide that is gotten from SOD’s action is metabolized by GPx to yield to water and oxygen using reduced glutathione as a hydrogen donor [26,29]. GST plays a vital function in the cellular detoxification of ROS by conjugating with GSH [29]. In the diabetic group, the observed increase of their liver, kidney and serum MDA together with their decreased liver, kidney and serum activity of SOD and GPx, GSH concentration as well as serum GST activity implies local and systemic oxidative stress in this group. In contrast, the decreased liver, kidney and serum MDA together with the increased liver, kidney and serum activity of SOD, GPx, GSH concentration and serum GST activity of the diabetic animals which took gallic acid, vitamin C, and their combined treatment implies attenuation of local and systemic oxidative stress by these interventions.

From our study, vitamin C supplementation was more effective in diminishing local and systemic oxidative stress than gallic acid supplementation. This was apparent from the attenuated kidney and serum MDA values of the diabetic rats which were intervened with vitamin C contrasted with the diabetic rats which were intervened with gallic acid. Similarly, the combined treatment was more successful in diminishing local and systemic oxidative stress than the single therapies. This became obvious from the decreased liver, kidney and serum MDA plus the raised serum SOD levels of the diabetic rats which received the combined treatment contrasted with those diabetic rats that took the single treatments.

NF-κB is a transcription molecule whose activation is stimulated by amongst other factors, oxidant stress and upon activation, it activates other inflammatory molecules including TNFα [30]. Previous investigators showed the contribution of NF-κB and TNF-α to the pathogenesis of DM and its complications [1]. Moreover, CRP is an acute phase protein which the liver produces in reaction to inflammation and whereas it is considered an indicator of acute inflammation, elevated CRP levels were also reported during chronic inflammation [31] and in diabetic settings [32]. Therefore, the exacerbated serum levels of NF-κB, TNFα and CRP in the diabetic animals indicate their systemic inflammation. Conversely, the decreased serum NF-κB, TNFα and CRP concentration of the diabetic animals which took gallic acid, vitamin C and the combined treatment, imply attenuation of systemic inflammation by these interventions.

Again, vitamin C was more effective in diminishing local and systemic inflammation than gallic acid. This was seen from the abolished liver and kidney NF-κB and TNF-α concentrations and the abolished serum NF-κB, TNF-α and CRP concentration of the diabetic animals which were given vitamin C as against the diabetic rats which received gallic acid. Similarly, the combined treatment was better in diminishing local and systemic inflammation than the single treatments. This was apparent from the greater reduction of liver and kidney NF-κB and TNF-α levels and greater reduction of serum NF-κB, TNF-α and CRP concentrations of the diabetic animals which took the combined treatment in comparison to the diabetic animals which were intervened with the single treatments.

Destruction of the liver cells causes intracellular enzymes (AST, ALT and ALP) to leak from the liver into the blood [33]. Thus, elevated serum activities of AST, ALT and ALP are taken as indicators of liver injury or pathology [33].

Therefore, the elevated serum AST, ALT and ALT activities and the decreased AST/ALT ratio of the diabetic rats, indicates hepatic injury or pathology in them. Conversely, the decreased serum AST, ALT and ALT activities together with the increased AST/ALT ratio of the diabetic animals which were intervened with gallic acid, vitamin C and the combined treatment are pointers to the hepatoprotective actions of the interventions. Our study also showed that the hepatoprotective action of the combined treatment surpassed those of the single treatments. This was pronounced from the decreased ALT activity but increased AST/ALT ratio of the rats with diabetes which received the combined treatment contrasted with the diabetic rats which received the single treatments.

Serum urea concentrations as well as those creatinine, uric acid, total proteins and albumin serve as indicators of renal function or nephrotoxicity. When renal function is impaired, serum urea concentrations as well as those of creatinine and uric acid become raised. In addition, during glomerular permeability, albumin and other plasma proteins are excreted in urine leading to decreased concentration of total proteins in circulation [33–35]. Further, a decline in total proteins and serum albumin could also stem from increased protein glycation [26]. Therefore, elevation of serum urea concentration as well as creatinine and uric acid together with the reduced serum total protein and albumin levels of the diabetic rats, indicates nephrotoxicity or nephropathology for these rats. The decreased serum urea concentration as well as creatinine and uric acid together with the increased serum total proteins and albumin concentrations of the diabetic animals which were intervened with gallic acid, vitamin C plus the combined treatment indicate the renal protective actions of these interventions. The renal protective action of the combined treatment was greater than the single therapies in this study. This was apparent from the decreased creatinine but increased albumin concentrations of the rats with diabetes which received the co-treatment contrasted with the diabetic rats which were given the single therapies.

The histopathological assessment of the liver and kidney of the diabetic rats showed degeneration of these tissues with severe aggregate of periportal inflammation, congestion of blood vessels and hemorrhage in the hepatic tissues and severe fatty changes with hemorrhagic and necrotic glomeruli and chronic nephritis for the renal tissues validate our biochemical findings. The revamped histology of the liver and kidney of the rats which were intervened with gallic acid, vitamin C as well as gallic acid plus vitamin C combined treatment, lend credence to our biochemical findings, on the hepatic and renal protective actions of these interventions.

However, vitamin C supplementation at the dose that was used in this study was better than gallic acid supplementation in ameliorating the histopathological changes in the liver and kidneys of the fructose/STZ induced type 2 diabetic animals whereas gallic acid/vitamin C combined treatment at the dose that was used was found to be more efficacious than the single therapies in ameliorating the histopathological changes in the liver and kidneys of type 2 diabetic rats brought on by fructose and STZ exposure.

A limitation of this present study was that we could not determine the molecular mechanisms of the hepatoprotective and renal protective effects of these interventions in this research. Given that rats are able to synthesize vitamin C, our study is also limited by our inability to use the right animal model (such as guinea pig) that does not synthesize vitamin C as usage of rats in this study may have influenced the vitamin C results that were obtained in this study.

In conclusion, the study revealed that combined treatment of gallic acid and vitamin C at the dose that was used, was better than gallic acid or vitamin C as single therapies in achieving glycemic control, attenuating systemic and local oxidative stress and inflammation as well as ameliorating histopathological changes in the liver and kidneys of fructose/STZ induced type 2 diabetes in rats.

## Figures and Tables

**Table 1:** Effect of gallic acid and vitamin C co-treatment on oxidative stress and antioxidant markers in the tissues of rats with T2DM.

*Parameters*	Control	Diabetic	Vitamin C	Gallic acid	GA + Vit C
*MDA (Liver)*	3.14±0.63	6.44±0.53#	4.76±0.57^a^	4.84±0.46^a^	4.04±0.40^a^,^b^,^c^
*SOD (Liver)*	49.59±7.49	28.68±6.36#	38.37 ±3.39^a^	36.11±4.42	40.65±5.71^a^
*GPx (Liver)*	4.45±0.33	2.14±0.51#	3.26±0.40^a^,^c^	2.69±0.45^a^	3.83±0.12^a^,^b^,^c^
*GSH (Liver)*	39.59±3.22	15.32±1.82#	26.31±2.79^a^,^c^	21.58±5.19^a^	31.53±3.12^a^,^b^,^c^
*MDA (Kidney)*	3.47±0.56	7.11±0.20#	4.82±0.41^a^,^c^	5.50±0.36^a^	4.09±0.61^a^,^b^,^c^
*SOD (Kidney)*	44.08±3.71	26.64±5.07#	32.84 ±3.73^a^	31.75±2.59^a^	33.08±2.64^a^
*GPx (Kidney)*	5.13±0.73	2.16±0.21#	3.92±0.52^a^	3.90±0.42^a^	4.32±0.33^a^
*GSH (Kidney)*	39.03±5.61	16.19±1.15#	27.11±2.76^a^,^c^	21.78±3.67^a^	32.73±4.88^a^,^b^,^c^

Results were reported as means ± standard deviation.

# statistically significant (P<0.05) when compared with the control.

a statistically significant (P<0.05) when compared with the diabetic;

b statistically significant (P<0.05) when compared with vitamin C;

c statistically significant (P<0.05) when compared with gallic acid.

MDA – Malondialdehyde (μmol/mg protein); SOD – Superoxide dismutase (units/mg protein); GPx – Glutathione peroxidase (units/mg protein); GSH – reduced glutathione (μmol/mg protein).

**Table 2:** Effect of gallic acid and vitamin C co-treatment on inflammatory markers in the tissues of rats with T2DM.

*Parameters*	Control	Diabetic	Vitamin C	Gallic acid	GA + Vit C
*NF-κB (Liver)*	21.84±2.04	48.35±4.21#	37.61±3.77^a^,^c^	43.64±1.33^a^	31.80±2.11^a^,^b^,^c^
*TNF-α (Liver)*	75.89±5.77	139.18±6.38#	114.48±7.34^a^,^c^	129.07±7.32^a^	100.26±2.88^a^,^b^,^c^
*NF-κB (Kidney)*	22.48±3.30	47.64±5.11#	35.77±4.73^a^,^c^	42.07±1.41^a^	28.80±4.30^a^,^b^,^c^
*TNF-α (Kidney)*	77.58±9.06	142.14±7.51#	117.76±5.24^a^,^c^	129.63±5.30^a^	105.93±9.94^a^,^b^,^c^

Results were reported as means ± standard deviation.

# statistically significant (P<0.05) when compared with the control.

a statistically significant (P<0.05) when compared with the diabetic;

b statistically significant (P<0.05) when compared with vitamin C;

c statistically significant (P<0.05) when compared with gallic acid.

NF-κB – Nuclear factor kappa B (pg/mg protein); TNF-α – Tumor necrosis factor alpha (pg/g protein).
